# Around the Hospitals

**Published:** 1894-05-05

**Authors:** 


### Around the Hospitals.

[Notice to the Managers of Institutions.—Special space is now reserved for the insertion of notices of hospital meetings and festivals. The Editor requests that all notices may reach The Hospital Office, 428, Strand, at least one week before the date of each meeting.]

**The Suffolk General Hospital.**—A good many improvements have been carried out at this institution, principally through the generosity of the Duke of Grafton and the donors to his fund, which resulted in a collection of £867. This was expended on: (1) A hot-water apparatus, by means of which the chapel and passages are now warmed; (2) a new lift; (3) the reparation of the outside of the building; and (4) a disinfecting tank and other appliances for the laundry. The tiling of the lavatories has also been effected during the year, apart from this fund. The committee, at the annual meeting, agreed to the admission of paying guests. One had already been received, before the passing of the rule, at a charge of £2 2s. per week. These paying patients are to be received not only for their own convenience, but also as benefiting the institution. For both reasons we would point out that £2 2s. is an unnecessarily low sum to ask of the well-to-do, to whom the cost of an illness at home would be considerably greater. As a rule people are only too apt to pay as little as they can for benefits received from hospitals, and the managers of these institutions in England seldom take pains that relief is given, free or otherwise, in just proportion to means. It would be well if all employers would follow the example of the gentleman who sent one of his domestic servants for treatment, and at the termination of the illness forwarded a cheque for £10 to the hospital. The well-to-do frequently apparently regard a subscription of a few guineas a year to a hospital sufficient reason for sending their employés for treatment without further payment on their behalf. During the year 490 in-patients and 1,575 out-patients were treated at the hospital. The accounts show a decrease of £80 on expenditure, and receipts an increase of £40. Some small legacies have been received, and one of £500, which is to be placed to the capital account, is yet to come.

